# Supplementary material for: The Complete Mitogenomes of Three Grasshopper Species with Special Notes on the Phylogenetic Positions of Some Related Genera
Source: Insects. 2023 Jan 13;14(1):85. doi: 10.3390/insects14010085 (PMC9865218; doi:10.3390/insects14010085)
Supplement: Supplementary file 1 [file insects-14-00085-s001.zip › Figure S1-S5.pdf]

-

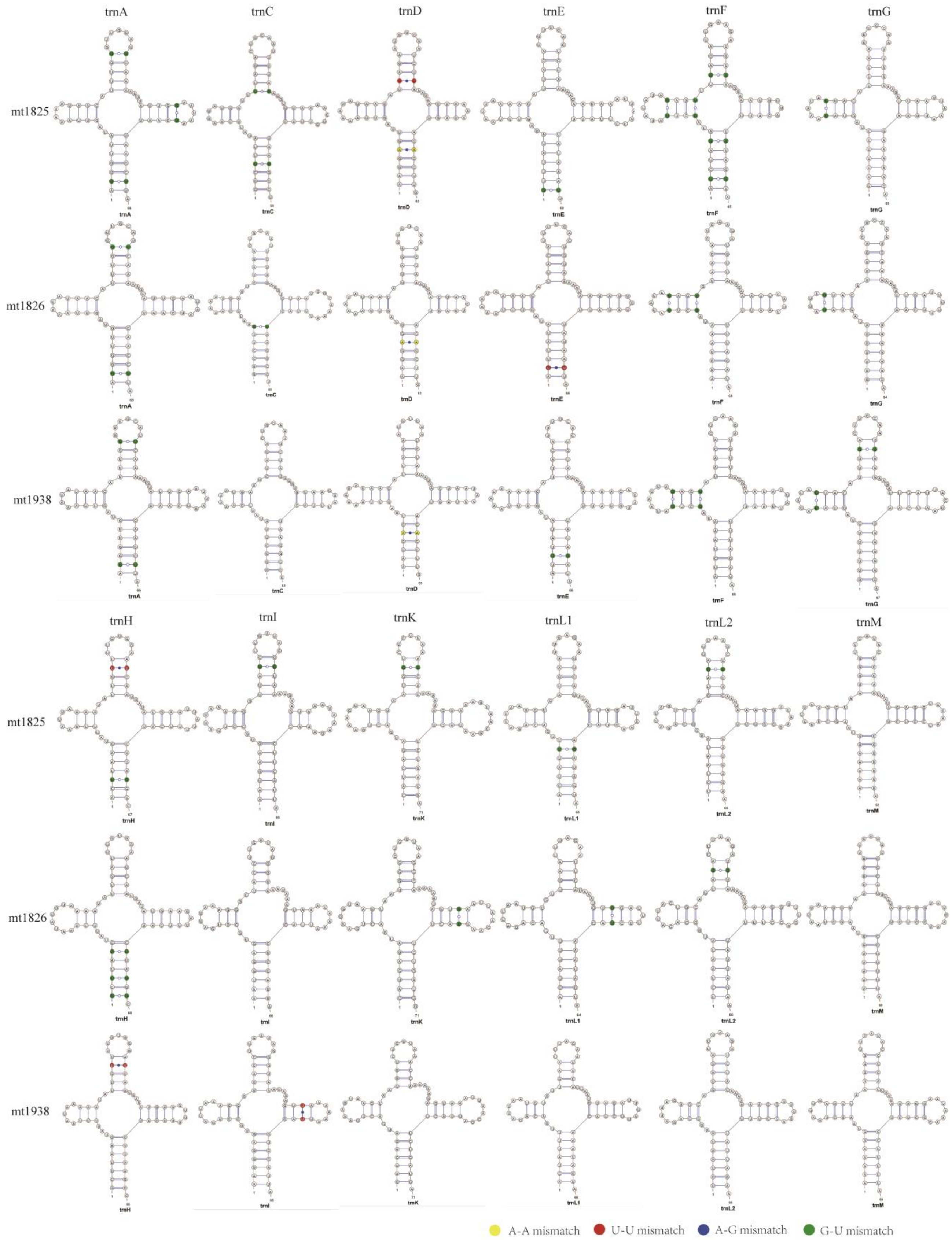

Figure S1. Secondary structures of 22 tRNAs of the three newly sequenced mitogenomes. mt1825: *Longzhouacris mirabilis*, mt1826: *Ranacris albicornis*, mt1938: *Conophyma zhaosuensis*.

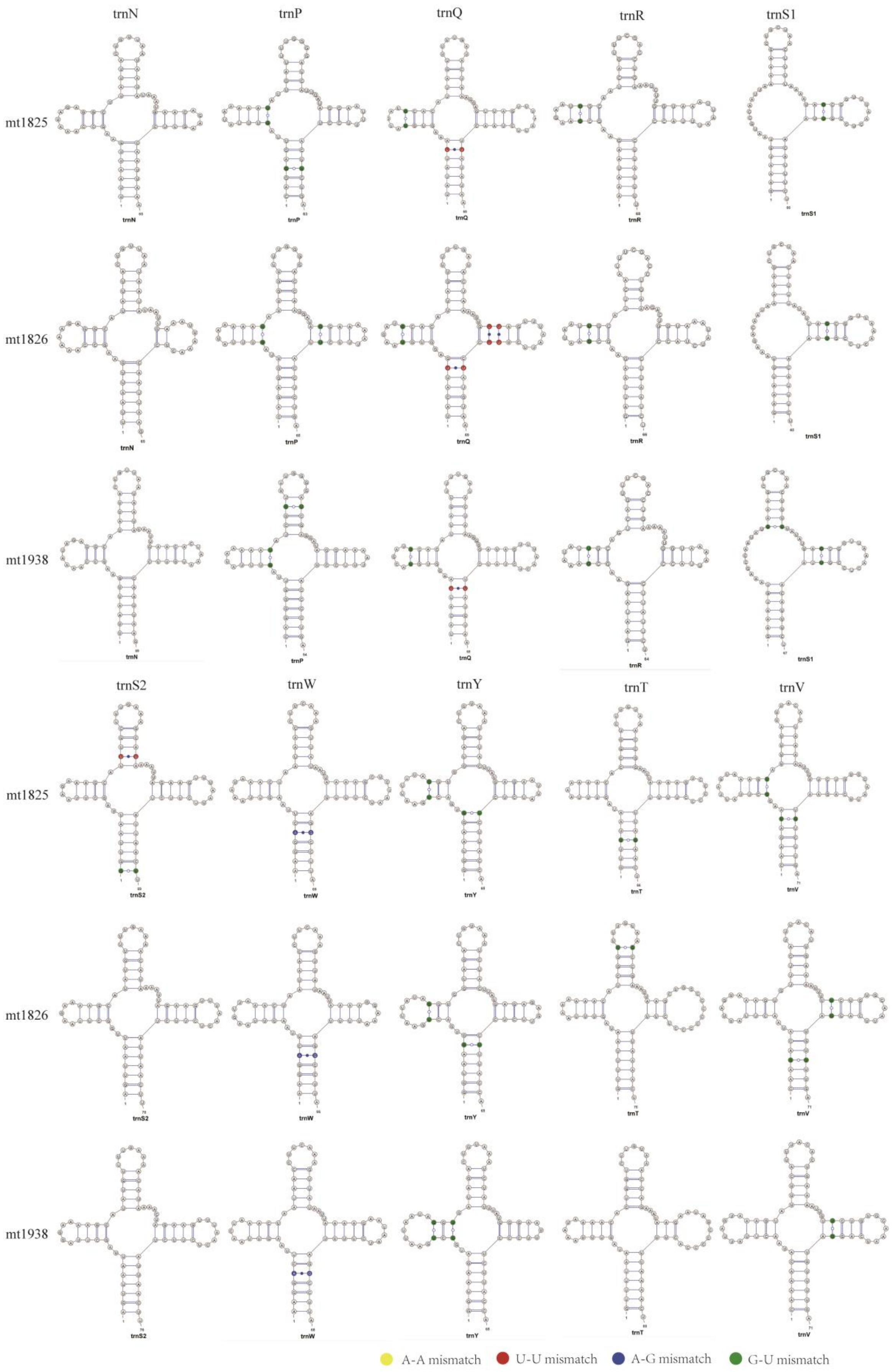

Figure S1. （continued）

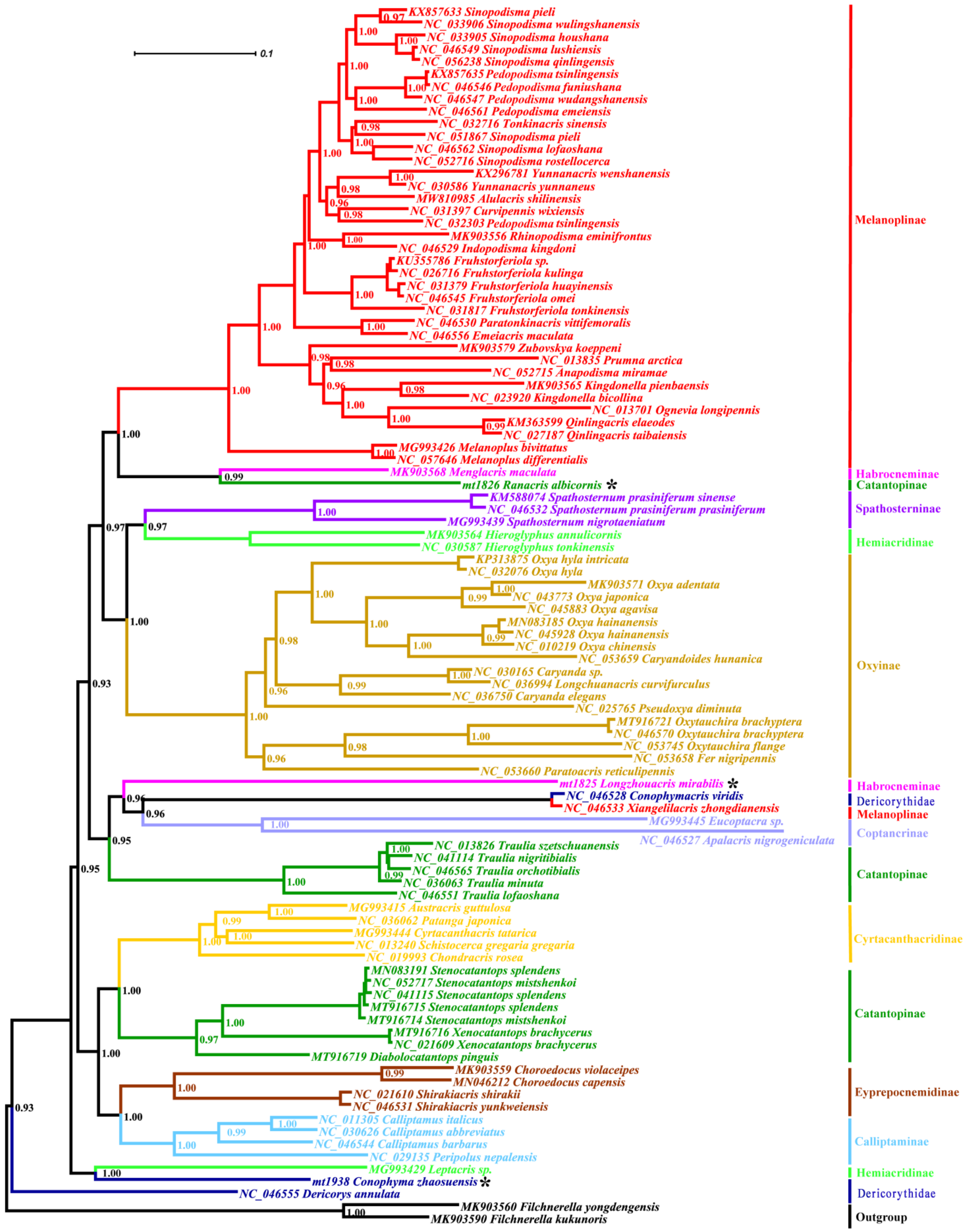

Figure S2. Phylogenetic tree reconstructed from sequences of the 13 mitochondrial PCGs using Bayesian inference. The asterisk indicates the three newly sequenced species.

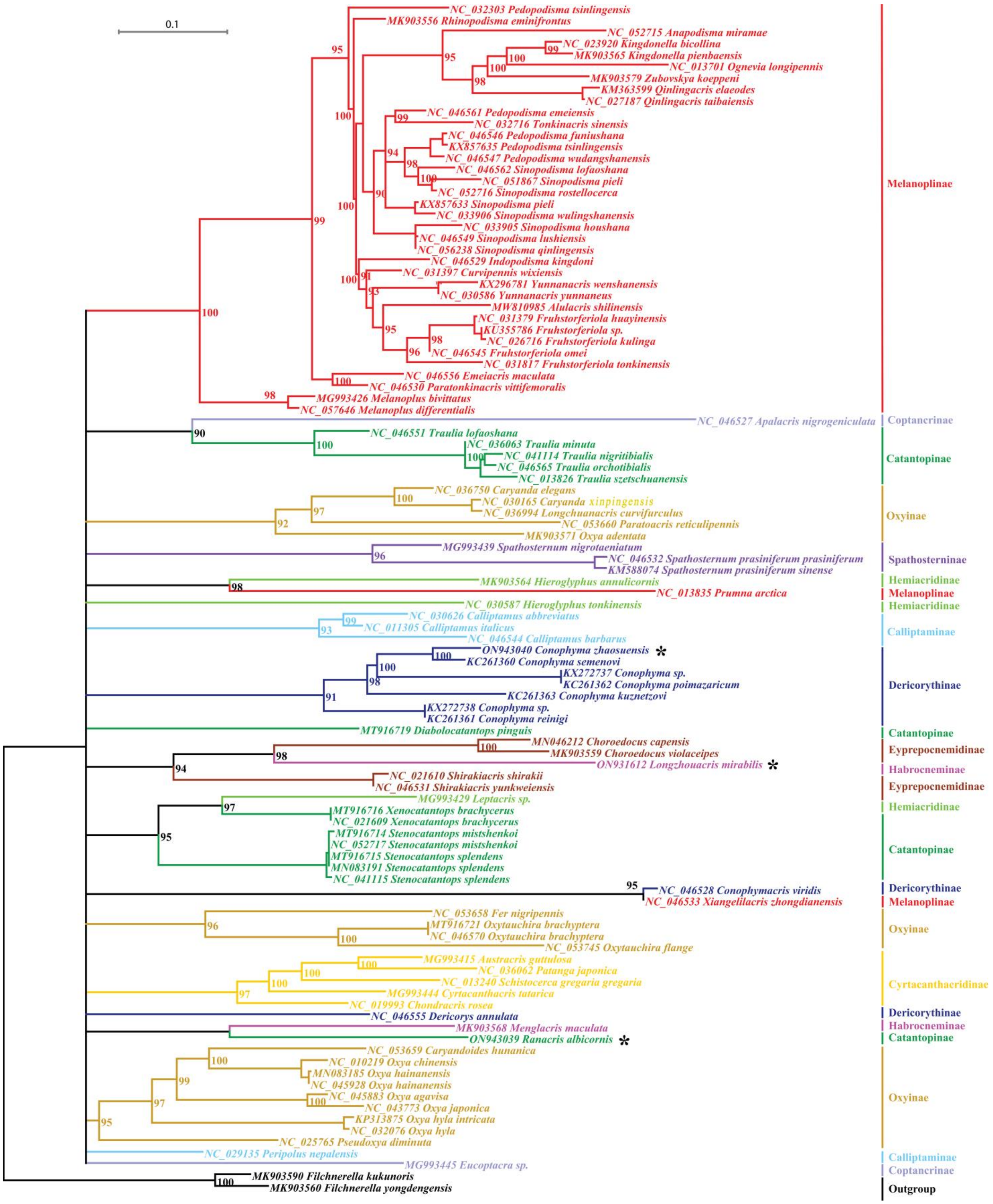

Figure S3. Phylogenetic tree reconstructed from partial sequences of the *COX1* gene using maximum likelihood. The asterisk indicates the three newly sequenced species.

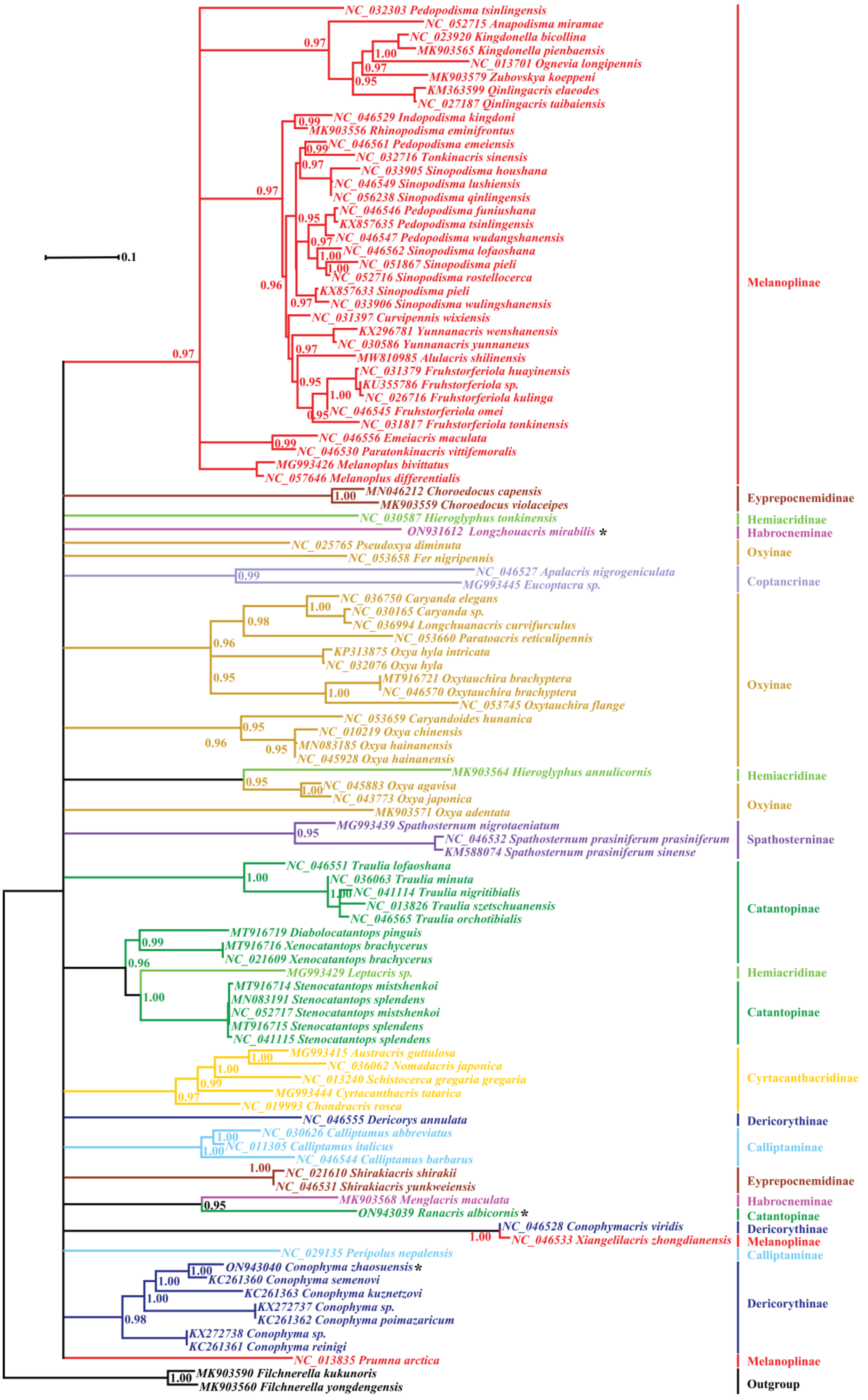

Figure S4. Phylogenetic tree reconstructed from partial sequences of the *COX1* gene using Bayesian inference. The asterisk indicates the three newly sequenced species.

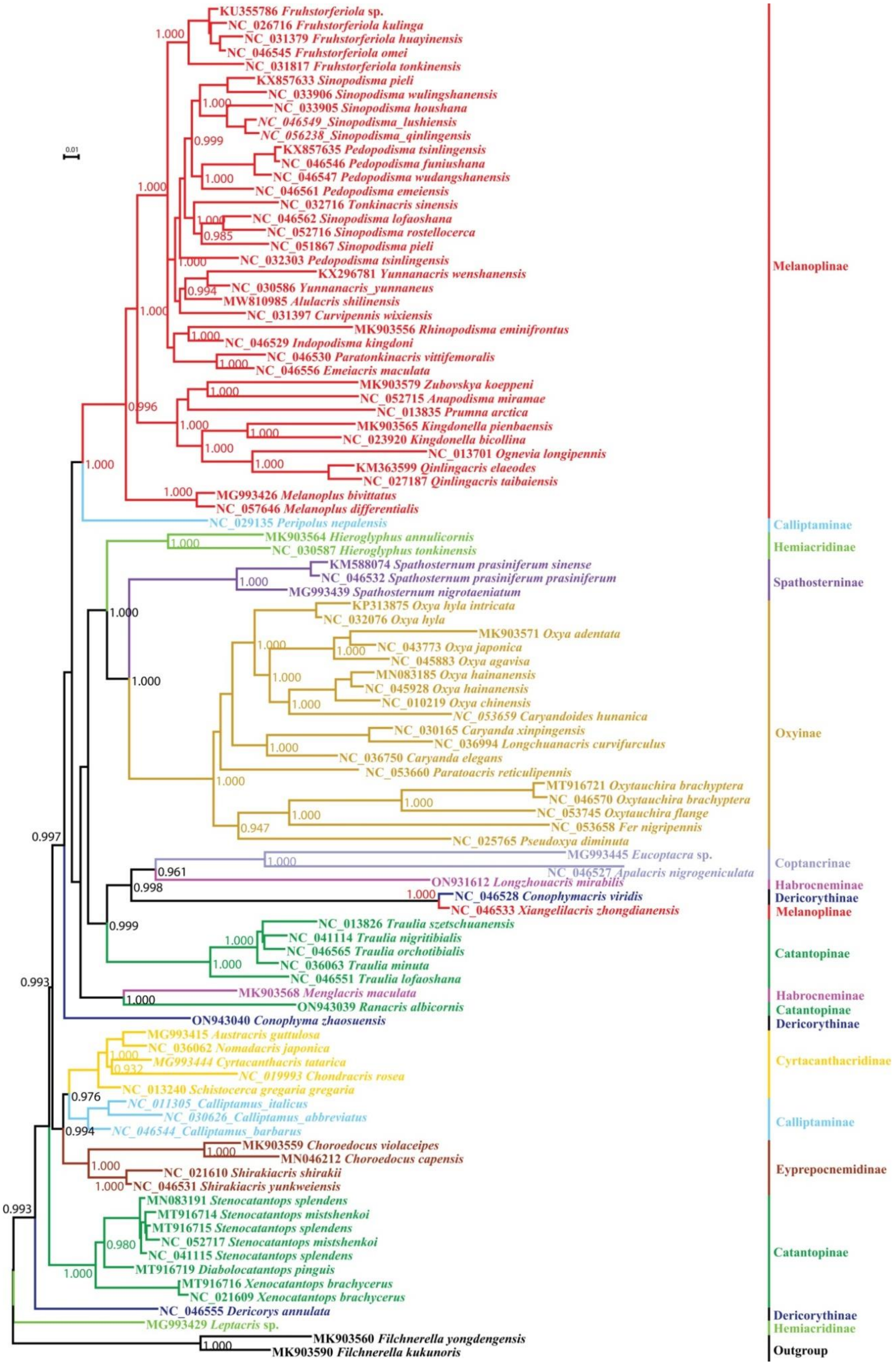

Figure S5. Phylogenetic tree reconstructed from amino acid sequences of the 13 mitochondrial PCGs gene using Bayesian inference with MtRev model. The asterisk indicates the three newly sequenced species.
