# Supplementary material for: The Complete Mitogenomes of Three Grasshopper Species with Special Notes on the Phylogenetic Positions of Some Related Genera
Source: Insects. 2023 Jan 13;14(1):85. doi: 10.3390/insects14010085 (PMC9865218; doi:10.3390/insects14010085)
Supplement: Supplementary file 1 [file insects-14-00085-s001.zip › TableS1-S10.pdf]

Table S1. Accession numbers and references of COI sequences of *Conophyma*spp. sampled in this study.

Species	Accession number	Reference
**Caelifera, Acridoidea, Dericorythidae Conophyminae**		
Conophyma kuznetzovi	KC261363	Bugrov *et al*., 2012
Conophyma poimazaricum	KC261362	Bugrov *et al*., 2012
Conophyma reinigi	KC261361	Bugrov *et al*., 2012
Conophyma semenovi	KC261360	Bugrov *et al*., 2012
Conophyma sp.	KX272737.	Direct Submission
Conophyma sp.	KX272738	Direct Submission

References

[102] Bugrov, A. G., Smyshlyaev, G. A., Blinov, A. G.. Phylogeny of grasshoppers (Orthoptera, Acrididae) based on the analysis of DNA sequences in COI mitochondrial gene. Euroasian Entomological Journal, 2012, 11: 493–502.

Table S2. The best–fitting models used for phylogenetic analyses of the mitochondrial PCGs dataset.

Information Criterion for Model Selection	Best Model	Partition Names
AICc	GTR+F+I+G4	*ND3*_codon2
	GTR+F+R3	*ND1*_codon2
	GTR+F+R4	*ND2*_codon1，*ND5*_codon2，*ND4*_codon3，*COX1*_codon1，*ND6*_codon3，*COX2*_codon1，*ND3*_codon1
	GTR+F+R5	*ND5*_codon3，*ND4*_codon1，*COX1*_codon3
	HKY+F+R3	*CYTB*_codon2，*COX3*_codon2
	HKY+F+R4	*ND4L*_codon1
	TIM2+F+R3	*COX3*_codon1，*ND3*_codon3
	TIM2+F+R4	*ND1*_codon3，*CYTB*_codon1，*ATP6*_codon1，*ND6*_codon2，*ND5*_codon1
	TIM3+F+I+G4	*ATP8*_codon3，*ATP8*_codon2
	TIM3+F+R2	*ATP6*_codon2
	TIM3+F+R3	*COX1*_codon2，*ND4L*_codon2
	TIM3+F+R4	*COX2*_codon3，*ND2*_codon2
	TN+F+R3	*ND6*_codon1
	TN+F+R4	*ND2*_codon3
	TN+F+R5	*CYTB*_codon3
	TPM2u+F+R4	*ND1*_codon1
	TPM2u+F+R5	*ATP6*_codon3
	TVM+F+I+G4	*ATP8*_codon1
	TVM+F+R3	COX2_codon2，ND4L_codon3
	TVM+F+R4	ND4_codon2，COX3_codon3
BIC	GTR+F+G4	*ND1*_codon1，*ND6*_codon3，*COX3*_codon3，*COX3*_codon1，*ND6*_codon1，*ND4*_codon1，*ND6*_codon2，*ND4L*_codon3，*ND4L*_codon2，*ND5*_codon1
	GTR+F+I+G4	*ND2*_codon1, *ND4*_codon2，*ND4*_codon3，*ND5*_codon2，*ND2*_codon3，*ND1*_codon3，*ND2*_codon2，*COX1*_codon1，*COX2*_codon1，*ATP6*_codon1，*COX1*_codon3，*COX1*_codon2，*CYTB*_codon1，*COX2*_codon2，*ATP6*_codon2，*ND3*_codon1，*ATP8*_codon2，*ATP8*_codon1
	HKY+F+G4	*ND3*_codon3，*ND4L*_codon1，*ATP8*_codon3
	HKY+F+I+G4	*ND5*_codon3，*COX2*_codon3，*ND1*_codon2，*CYTB*_codon3，*ATP6*_codon3，*CYTB*_codon2，*COX3*_codon2，*ND3*_codon2

Table S3. The best–fitting models used for the phylogenetic analyses of the partial *COX1* dataset.

Information Criterion for Model Selection	Best Model	Partition Names
AICc	SYM+I+G4	*COX1*_codon1
	TN+F+R6	*COX1*_codon2
	TPM2u+F+R4	*COX1*_codon3
BIC	SYM+I+G4	*COX1*_codon1
	GTR+F+I+G4	*COX1*_codon2
	HKY+F+G4	*COX1*_codon3

Table S4. Nucleotide composition of the mitogenome of *Longzhouacris mirabilis* (mt1825).

Feature	Length(bp)	T%	C%	A%	G%	A+T%	AT-skew	GC-skew
Whole genome	16164	32.9	14.2	42.3	10.5	75.2	0.1250	-0.1498
Protein-coding genes	11148	32.4	14.4	42.0	11.1	74.4	0.1290	-0.1294
First codon position	3716	29.8	11.4	45.8	13.0	75.6	0.2116	0.0656
Second codon position	3716	34.5	19.5	31.1	14.9	65.6	-0.0518	-0.1337
Third codon position	3716	33.0	12.3	49.2	5.5	82.2	0.1971	-0.3820
Protein-coding genes-J	6864	37.0	14.7	36.2	12.1	73.2	-0.0109	-0.0970
First codon position	2288	30.0	13.8	36.7	19.4	66.7	0.1004	0.1687
Second codon position	2288	44.2	21.9	20.1	13.7	64.3	-0.3748	-0.2303
Third codon position	2288	36.6	8.4	51.7	3.2	88.3	0.1710	-0.4483
Protein-coding genes-N	4284	25.2	13.9	51.4	9.5	76.6	0.3420	-0.1880
First codon position	1428	29.4	7.5	60.4	2.7	89.8	0.3452	-0.4706
Second codon position	1428	19.0	15.5	48.7	16.7	67.7	0.4387	0.0373
Third codon position	1428	27.2	18.6	45.1	9.1	72.3	0.2476	-0.3430
ATP6	678	38.2	14.6	37.9	9.3	76.1	-0.0039	-0.2218
ATP8	159	38.4	13.8	42.8	5.0	81.2	0.0542	-0.4681
COX1	1540	34.7	16.7	33.8	14.8	68.5	-0.0131	-0.0603
COX2	684	35.1	15.4	37.6	12.0	72.7	0.0344	-0.1241
COX3	792	36.2	15.2	34.5	14.1	70.7	-0.0240	-0.0375
CYTB	1140	40.7	13.2	33.4	12.7	74.1	-0.0985	-0.0193
ND1	945	25.4	14.0	49.9	10.7	75.3	0.3254	-0.1336
ND2	1020	36.3	14.7	37.9	11.1	74.2	0.0216	-0.1395
ND3	354	36.2	14.4	39.0	10.5	75.2	0.0372	-0.1566
ND4	1336	24.8	14.2	52.2	8.8	77.0	0.3558	-0.2348
ND4L	294	21.8	12.2	57.5	8.5	79.3	0.4502	-0.1787
ND5	1719	26.2	13.8	50.5	9.5	76.7	0.3168	-0.1845
ND6	522	39.1	10.7	41.6	8.6	80.7	0.0310	-0.1088
rrnL	1389	33.6	14.2	43.7	8.5	77.3	0.1307	-0.2511
rrnS	792	31.3	16.5	42.3	9.8	73.6	0.1495	-0.2548
D - loop	1348	36.2	12.3	44.5	7.0	80.7	0.1029	-0.2746

Table S5. Nucleotide composition of the mitogenome of *Ranacris albicornis* (mt1826).

Feature	Length(bp)	T%	C%	A%	G%	A+T%	AT-skew	GC-skew
Whole genome	15720	33.1	14.2	42.0	10.7	75.1	0.1185	-0.1406
Protein-coding genes	11148	33.1	14.3	41.6	11.0	74.7	0.1138	-0.1304
First codon position	3716	30.4	11.3	44.9	13.4	75.3	0.1926	0.0850
Second codon position	3716	34.6	19.6	31.0	14.7	65.6	-0.0549	-0.1429
Third codon position	3716	34.4	11.8	49.0	4.8	83.4	0.1751	-0.4217
Protein-coding genes-J	6867	37.2	14.8	36.1	11.9	73.3	-0.0150	-0.1086
First codon position	2289	29.0	14.7	36.3	20.1	65.3	0.1118	0.1552
Second codon position	2289	44.3	22.1	20.4	13.2	64.7	-0.3694	-0.2521
Third codon position	2289	38.3	7.6	51.7	2.5	90.0	0.1489	-0.5050
Protein-coding genes-N	4281	26.7	13.4	50.5	9.4	77.2	0.3083	-0.1754
First codon position	1427	32.7	5.9	58.7	2.7	91.4	0.2845	-0.3721
Second codon position	1427	19.2	15.6	48.1	17.2	67.3	0.4294	0.0488
Third codon position	1427	28.2	18.7	44.7	8.4	72.9	0.2263	-0.3801
ATP6	678	37.3	15.3	38.2	9.1	75.5	0.0119	-0.2541
ATP8	162	37.7	13.6	44.4	4.3	82.1	0.0816	-0.5196
COX1	1540	35.5	15.9	33.4	15.1	68.9	-0.0305	-0.0258
COX2	684	35.1	14.2	37.6	13.2	72.7	0.0344	-0.0365
COX3	792	36.7	15.5	33.6	14.1	70.3	-0.0441	-0.0473
CYTB	1137	40.7	13.5	33.7	12.1	74.4	-0.0941	-0.0547
ND1	945	26.3	13.2	49.6	10.8	75.9	0.3070	-0.1000
ND2	1023	35.0	16.4	38.3	10.3	73.3	0.0450	-0.2285
ND3	354	36.4	15.5	38.1	9.9	74.5	0.0228	-0.2205
ND4	1335	26.7	13.9	50.5	8.8	77.2	0.3083	-0.2247
ND4L	294	22.8	11.2	56.8	9.2	79.6	0.4271	-0.0980
ND5	1719	27.7	13.4	49.7	9.1	77.4	0.2842	-0.1911
ND6	522	42.0	9.4	41.6	7.1	83.6	-0.0048	-0.1394
rrnL	1382	33.7	14.5	42.7	9.1	76.4	0.1178	-0.2288
rrnS	806	32.0	15.9	41.8	10.3	73.8	0.1328	-0.2137
D－loop	860	31.7	11.7	49.2	7.3	80.9	0.2163	-0.2316

Table S6. Nucleotide composition of the mitogenome of *Conophyma zhaosuensis* (mt1938).

Feature	Length(bp)	T%	C%	A%	G%	A+T%	AT-skew	GC-skew
Whole genome	16190	32.1	14.3	42.5	11.2	74.6	0.1394	-0.1216
Protein-coding genes	11175	42.3	12.8	31.9	13.1	74.2	-0.1402	0.0116
First codon position	5397	30.3	15	41.7	13	72	0.1583	-0.0714
Second codon position	5397	32.5	14.3	43.2	9.9	75.7	0.1413	-0.1818
Third codon position	5396	33.4	13.5	42.5	10.6	75.9	0.1199	-0.1203
Protein-coding genes-J	6880	36.9	14.6	35.8	12.7	72.7	-0.0151	-0.0696
First codon position	2294	35.8	11.5	41.7	11	77.5	0.0761	-0.0222
Second codon position	2293	36.1	17.2	30.2	16.5	66.3	-0.0890	-0.0208
Third codon position	2293	38.8	15.2	35.4	10.6	74.2	-0.0458	-0.1783
Protein-coding genes-N	4295	50.8	9.8	25.7	13.7	76.5	-0.3281	0.1660
First codon position	1432	52.7	6.6	28.6	12.1	81.3	-0.2964	0.2941
Second codon position	1432	45.5	12.8	23.6	18.1	69.1	-0.3169	0.1715
Third codon position	1431	54.2	9.9	25	10.9	79.2	-0.3687	0.0481
ATP6	678	38.3	14.3	38.2	9.1	76.5	-0.0013	-0.2222
ATP8	162	36.4	12.3	46.9	4.3	83.3	0.1261	-0.4819
COX1	1531	35.6	15.8	32.9	15.7	68.5	-0.0394	-0.0032
COX2	684	33	15.1	37.9	14	70.9	0.0691	-0.0378
COX3	792	37.4	15.8	31.8	15	69.2	-0.0809	-0.0260
CYTB	1137	38.2	14.7	33.9	13.2	72.1	-0.0596	-0.0538
ND1	945	25.2	13.5	50.9	10.4	76.1	0.3377	-0.1297
ND2	1021	36	15.2	37.1	11.7	73.1	0.0150	-0.1301
ND3	354	37.3	13.3	37.6	11.9	74.9	0.0040	-0.0556
ND4	1339	24.8	15	50	10.2	74.8	0.3369	-0.1905
ND4L	294	20.7	12.2	58.2	8.8	78.9	0.4753	-0.1619
ND5	1717	27.6	13	50.1	9.3	77.7	0.2896	-0.1659
ND6	521	42.2	9.6	40.7	7.5	82.9	-0.0181	-0.1228
rrnL	1365	32.1	14.4	45	8.6	77.1	0.1673	-0.2522
rrnS	796	31.2	16.7	40.8	11.3	72	0.1333	-0.1929
D - loop	1437	24.6	11.4	47	8.1	71.6	0.3128	-0.1692

Table S7. Codon usage of the PCGs of *Longzhouacris mirabilis* (mt1825).

Amino Acid	Codon	No.	RSCU(%)	Amino Acid	Codon	No.	RSCU(%)
Phe	UUU	313	1.58	Tyr	UAU	194	1.79
	UUC	84	0.42		UAC	23	0.21
Leu	UUA	336	4.30	End	UAA	37	1.48
	UUG	36	0.46		UAG	13	0.52
Leu	CUU	42	0.54	His	CAU	52	1.58
	CUC	7	0.09		CAC	14	0.42
	CUA	42	0.54	Gln	CAA	52	1.82
	CUG	6	0.08		CAG	5	0.18
Ile	AUU	326	1.73	Asn	AAU	177	1.60
	AUC	51	0.27		AAC	44	0.40
Met	AUA	190	1.72	Lys	AAA	64	1.31
	AUG	31	0.28		AAG	34	0.69
Val	GUU	65	1.68	Asp	GAU	54	1.74
	GUC	9	0.23		GAC	8	0.26
	GUA	74	1.91	Glu	GAA	64	1.71
	GUG	7	0.18		GAG	11	0.29
Ser	UCU	84	1.85	Cys	UGU	55	1.62
	UCC	24	0.53		UGC	13	0.38
	UCA	115	2.53	Trp	UGA	95	1.41
	UCG	9	0.20		UGG	40	0.59
Pro	CCU	56	1.75	Arg	CGU	15	1.22
	CCC	12	0.38		CGC	1	0.08
	CCA	56	1.75		CGA	31	2.53
	CCG	4	0.13		CGG	2	0.16
Thr	ACU	62	1.34	Ser	AGU	34	0.75
	ACC	27	0.58		AGC	15	0.33
	ACA	92	1.99		AGA	67	1.48
	ACG	4	0.09		AGG	15	0.33
Ala	GCU	47	1.49	Gly	GGU	71	1.46
	GCC	8	0.25		GGC	9	0.18
	GCA	71	2.25		GGA	97	1.99
	GCG	0	0.00		GGG	18	0.37

Table S8. Codon usage of the PCGs of *Ranacris albicornis* (mt1826).

Amino Acid	Codon	No.	RSCU(%)	Amino Acid	Codon	No.	RSCU(%)
Phe	UUU	285	1.75	Tyr	UAU	165	1.86
	UUC	41	0.25		UAC	12	0.14
Leu	UUA	375	4.23	End	UAA	0	0
	UUG	27	0.3		UAG	0	0
Leu	CUU	66	0.74	His	CAU	54	1.59
	CUC	5	0.06		CAC	14	0.41
	CUA	53	0.6	Gln	CAA	61	1.88
	CUG	6	0.07		CAG	4	0.12
Ile	AUU	358	1.86	Asn	AAU	141	1.67
	AUC	26	0.14		AAC	28	0.33
Met	AUA	250	1.84	Lys	AAA	81	1.56
	AUG	22	0.16		AAG	23	0.44
Val	GUU	86	1.86	Asp	GAU	63	1.7
	GUC	9	0.19		GAC	11	0.3
	GUA	85	1.84	Glu	GAA	70	1.69
	GUG	5	0.11		GAG	13	0.31
Ser	UCU	110	2.46	Cys	UGU	42	1.91
	UCC	12	0.27		UGC	2	0.09
	UCA	126	2.82	Trp	UGA	92	1.82
	UCG	3	0.07		UGG	9	0.18
Pro	CCU	59	1.71	Arg	CGU	17	1.24
	CCC	12	0.35		CGC	1	0.07
	CCA	63	1.83		CGA	34	2.47
	CCG	4	0.12		CGG	3	0.22
Thr	ACU	66	1.34	Ser	AGU	23	0.51
	ACC	18	0.37		AGC	1	0.02
	ACA	110	2.23		AGA	82	1.83
	ACG	3	0.06		AGG	1	0.02
Ala	GCU	75	1.81	Gly	GGU	103	1.89
	GCC	14	0.34		GGC	6	0.11
	GCA	76	1.83		GGA	92	1.69
	GCG	1	0.02		GGG	17	0.31

Table S9. Codon usage of the PCGs of *Conophyma zhaosuensis* (mt1938).

Amino Acid	Codon	No.	RSCU(%)	Amino Acid	Codon	No.	RSCU(%)
Phe	UUU	316	1.54	Tyr	UAU	155	1.7
	UUC	95	0.46		UAC	27	0.3
Leu	UUA	337	4.03	End	UAA	19	1.06
	UUG	46	0.55		UAG	17	0.94
Leu	CUU	59	0.71	His	CAU	52	1.53
	CUC	10	0.12		CAC	16	0.47
	CUA	48	0.57	Gln	CAA	59	1.87
	CUG	2	0.02		CAG	4	0.13
Ile	AUU	325	1.81	Asn	AAU	173	1.67
	AUC	35	0.19		AAC	34	0.33
Met	AUA	183	1.69	Lys	AAA	73	1.4
	AUG	34	0.31		AAG	31	0.6
Val	GUU	83	1.98	Asp	GAU	54	1.66
	GUC	5	0.12		GAC	11	0.34
	GUA	77	1.83	Glu	GAA	65	1.86
	GUG	3	0.07		GAG	5	0.14
Ser	UCU	80	1.84	Cys	UGU	46	1.39
	UCC	15	0.35		UGC	20	0.61
	UCA	117	2.7	Trp	UGA	99	1.51
	UCG	10	0.23		UGG	32	0.49
Pro	CCU	54	1.74	Arg	CGU	14	1.12
	CCC	7	0.23		CGC	0	0
	CCA	62	2		CGA	34	2.72
	CCG	1	0.03		CGG	2	0.16
Thr	ACU	53	1.11	Ser	AGU	28	0.65
	ACC	15	0.31		AGC	9	0.21
	ACA	116	2.43		AGA	74	1.71
	ACG	7	0.15		AGG	14	0.32
Ala	GCU	63	1.66	Gly	GGU	64	1.28
	GCC	8	0.21		GGC	6	0.12
	GCA	80	2.11		GGA	107	2.14
	GCG	1	0.03		GGG	23	0.46

Table S10. Annotation of the three newly sequenced complete mitogenomes.

**Name**	**Type**	Direction	Integenic nucleotides			Length		
			mt1825	mt1826	mt1938	mt1825	mt1826	mt1938
**trnI**	**tRNA**	forward	0	3	4	66	66	65
trnQ	tRNA	reverse	-1	10	7	69	69	69
trnM	tRNA	forward	0	0	0	68	69	69
ND2	CDS	forward	-2	-2	0	1020	1023	1021
trnW	tRNA	forward	-8	-8	-8	69	66	68
trnC	tRNA	reverse	9	5	7	64	68	63
trnY	tRNA	reverse	-8	-8	1	65	65	65
COX1	CDS	forward	0	0	0	1540	1540	1531
trnL2	tRNA	forward	10	7	7	66	66	66
COX2	CDS	forward	-2	-2	-2	684	684	684
trnD	tRNA	forward	2	2	2	63	63	65
trnK	tRNA	forward	14	18	-16	71	71	101
ATP8	CDS	forward	-7	-7	-7	159	162	162
ATP6	CDS	forward	4	3	4	678	678	678
COX3	CDS	forward	6	2	2	792	792	792
trnG	tRNA	forward	0	0	0	65	64	67
ND3	CDS	forward	0	-1	-1	354	354	354
trnA	tRNA	forward	1	5	4	66	65	66
trnR	tRNA	forward	5	6	0	68	66	64
trnN	tRNA	forward	0	0	0	65	65	69
trnS1	tRNA	forward	2	0	2	66	65	67
trnE	tRNA	forward	-1	0	-27	69	66	93
trnF	tRNA	reverse	0	2	1	65	64	66
ND5	CDS	reverse	15	15	15	1719	1719	1717
trnH	tRNA	reverse	3	4	-1	67	68	66
ND4	CDS	reverse	-7	-7	-7	1336	1335	1339
ND4L	CDS	reverse	2	2	2	294	294	294
trnT	tRNA	forward	0	0	0	66	70	69
trnP	tRNA	reverse	2	2	2	63	65	64
ND6	CDS	forward	8	3	8	522	522	521
CYTB	CDS	forward	-5	10	8	1140	1137	1140
trnS2	tRNA	forward	21	19	21	69	70	70
ND1	CDS	reverse	3	3	3	945	945	945
trnL1	tRNA	reverse	-59	-50	-42	65	64	66
rrnL	rRNA	reverse	-19	-19	-3	1389	1382	1365
trnV	tRNA	reverse	1	1	2	71	71	71
rrnS	rRNA	reverse	0	3	29	792	806	796
A+T	unsure	forward				1348	860	1437
